# Evaluation of the effects of three natural products and a hemostatic agent on wound healing: an experimental study

**DOI:** 10.55730/1300-0144.5558

**Published:** 2022-08-30

**Authors:** Ayşe ÇELİK YILMAZ, Dilek AYGİN

**Affiliations:** Department of Surgical Nursing, Faculty of Health Science, Sakarya University, Sakarya, Turkey

**Keywords:** Ankaferd blood stopper, propolis, silk protein, sweetgum oil, the experimental model, wound healing

## Abstract

**Background/aim:**

People have used many natural materials such as plant leaves, roots, liquids derived from plants, and animal products to treat wounds throughout history. It can be said that the research on wound care in recent years have focused on traditional and natural products again. This study aimed to investigate the effects of sweetgum oil, propolis, silk protein, and Ankaferd Blood Stopper (ABS) on wound healing in an experimental excisional wound model.

**Materials and methods:**

Including 36 Balb/c inbreed mice in the study were divided equally into four groups. Two circular excisional wounds were created on the dorsal skin of mice under anesthesia using a punch biopsy device. The wounds of the first group of mice were topically dressed with sweetgum oil, the second group mice with propolis, the third group mice with silk protein, and the fourth group mice with ABS daily. Tissue samples were taken from the wounds of mice on the 7th and 14th day of wound formation, and histological examinations were performed. On the 14th day, the wounds created in all mice were healed, and the experiment was terminated.

**Results:**

Mice in the silk protein group had faster wound healing. There was no statistical difference between the groups in immunohistochemical examinations. In the ABS group, the findings of the inflammatory process were more prominent.

**Conclusion:**

In conclusions, propolis, sweetgum oil, silk protein, and ABS positively affect different parameters in wound healing and support wound healing.

## 1. Introduction

Although there are many definitions of the wound in the literature, wound in general meaning; “the disruption of the anatomical and physiological integrity of the skin or mucosal surfaces due to any internal or external factor.” Wound healing is a well-organized repair process that begins after tissue damage. The healing process, which requires integrating different cells locally and systemically, aims to restore the tissue or organ to their anatomical, physiological, and functional integrity. The same events occur in the same order during the healing process, regardless of the tissue type or the cause of the injury. The interruption or prolonged duration of any of the healing phases causes a delay in wound healing, or the wound becomes chronic [1–4].

Wound is one of the health problems that human beings have been struggling with for centuries. In the historical process, materials in nature have been used frequently to close wounds. Examples of these are plant roots, seeds, leaves and extracts, animal fats, tissues such as wool, cotton, silk, various fibers, honey, milk, soil, and mud [5]. With the development of modern medicine, especially in the 19th century and after, biomedical products were developed, and their use in mass production became widespread. With these developments, significant advances have been made in wound care and infection prevention. With the new products developed, the use of natural materials in wound care has decreased. Despite many types of research on the pathophysiology of wound healing and new methods to support healing, still delayed and nonhealing wounds and complications pose a significant problem for both patients and healthcare professionals [6].

For this reason, researches on wound treatment are continuing intensively today. In the scientific world, in recent years, it has started to turn to natural products for wound care. This situation is founded on a cost-effective product that accelerates the wound healing process and has no side effects.

Due to its complex structure, wound healing is in the interest of many disciplines in health. Wound treatment is a field that nursing has been interested in since the past. Care should be given to nursing care based on careful and scientific research for wound healing. It should reach the evidence to be created for evidence-based care with the most up-to-date technological and scientific methods. In this regard, it is inevitable for nurses to engage in experimental research to develop biocompatible, nonside effects, and cost-effective products that can be used in wound care. Animal experiments have an important place in health professionals’ research to develop the ideal care product for wounds. Newly developed products can be tested on animals with preclinical studies before being administered to humans [6,7].

This research was carried out to investigate the effects of sweetgum oil (Liquidambar Orientalis), propolis, silk protein, and Ankaferd Blood Stopper (ABS) on acute wound healing. In the study, these products were applied topically to acute wounds. The relationship between inflammation, reepithelialization, granulation, angiogenesis, collagen synthesis, wound closure, and healing time was investigated.

## 2. Materials and methods

This preclinical, in vivo experimental, and prospective study was conducted at Sakarya University Experimental Medicine Application and Research Center (SUDETAM), between February and April 2020, after obtaining Sakarya University Faculty of Medicine Animal Experiments Local Ethics Committee approval. The study sample consisted of 36 standard BALB/c mice, weaned, 12–24 weeks old, weighing 25 ± 3 g, obtained from SUDETAM. The resulting mice were housed adlibutum with water and standard commercial feed, well ventilated, at a relative humidity of 60%, at 22 ± 1 °C, on a 12-h day/night cycle. The number of animals to be used in the study was determined by G power analysis. For statistical power analysis, when the Type 1 Error Alpha: 0.05, the power of the study was 0.80 (power), and the effect size of 0.55 (using Cohen’s criteria) was taken as significant, the high difference between the groups was taken, the minimum sample size required for the study was 36 (equal in the groups’ number of nine mice). Before the surgical procedure, the mice were anesthetized. After anesthesia, the back hair of the mice was shaved, and local field cleaning was performed. In the following process, two circular wounds were created for each mouse. Dressing products were applied topically to the wounds daily. The natural products in the experiment were used in standardized forms suitable for use in the wound. Photographs of each wound were taken daily, and the size of the wound was measured for the follow-up of the wound healing stages. In addition, histochemical examinations were performed on tissue samples taken from the wound area on the 7th and 14th days.

### 2.1. Histopathological examination

For histopathological examination, skin tissue samples taken from the wound area were fixed in a 10% buffered formaldehyde solution for 72 h. As part of the routine follow-up process; dehydration was achieved by passing tissue samples through alcohol series. It was passed through the xylol series to make it transparent and then blocked in paraffin. Sections of 4μm thickness taken from these blocks on the microtome (Leica RM 2135) were stained according to the hematoxylin-eosin (HE) staining technique and examined under a light microscope. Immunohistochemistry was performed to mark IL-6, IGF, EGF, Ki-67, and hydroxyproline at the wound site.

### 2.2. Statistical analysis

In the study, the data collected by observational and immunohistochemical methods were evaluated by transferring them to the IBM SPSS Statistics 23 program. In the normality analyses performed on the data obtained from the research, it was seen that it was suitable for normal distribution, and parametric tests were used in the study of the data. In the normality analysis of the data, kurtosis value (0.083) and skewness measure (0.887) were taken as a basis [8,9]. Frequency distribution (number, percentage) for categorical variables and descriptive statistics (mean, standard deviation) for numerical variables are given. The dependent sample t-test was used for the difference between two times, and the Repeated Measures ANOVA test was used for the difference between more than two times. One-way analysis of variance (one way ANOVA) was used to determine whether there was a difference between more than two groups. As a result of ANOVA, firstly Levene test for homogeneity of variance, and then from which group or groups the difference originated was checked with the “multiple comparison test” (Bonferroni). P < 0.05 was accepted for significance.

## 3. Results

**Figure 1:**
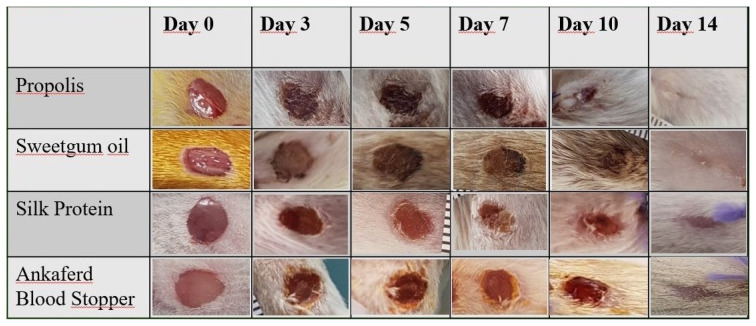
Wound photographs of a randomly selected mouse from the groups.

In the initial phase of the experiment, the weight of the mice was measured, and their average weight was found to be 20.97 g (min: 18.2, max: 28.1 ± 2.6). Weight measurements were not repeated in the rest of the experiment, as no change was made in the diet of the mice, and no diabetes model was used in the experiment. Wounds created in mice were observed during daily wound care; bleeding, inflammation, irritation, or infection of the aspects were evaluated. Wound inflammation and infection temperature increase that the findings, redness, swelling, discharge, etc., no evidence. No irritation developed in the healthy skin and wound area due to the products used in wound care. The halves were photographed every day due to the insignificance of daily changes; the photographs of the 0th, 3rd, 5th, 7th, 10th, and 14th days and the wound sizes were evaluated. Images of the wounds of one mouse from each group are given in Figure 1.

As a result of the Repeated Measures ANOVA test applied, there was a statistically significant difference between the times in terms of wound size in the groups (p = 0.044). Accordingly, it can be said that the mean wound size decreased significantly for each group over time (Table 1).

**Figure 2:**
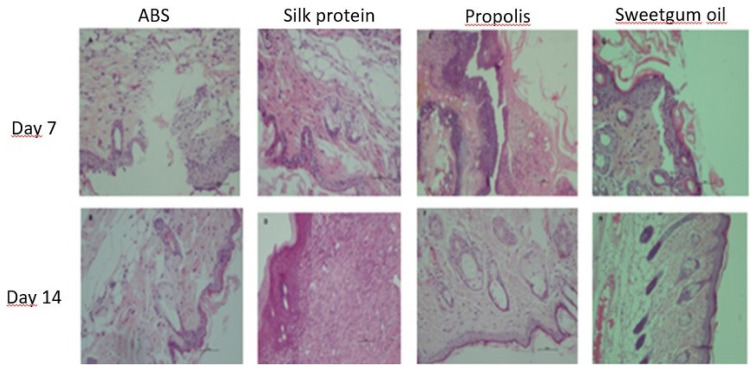
Hematoxylin eosin dye preparations belonging to groups (50 scale bar at 200X magnification)

Since an acute wound was created in the study, damage caused by substantial scar tissue was not found in histological sections. However, the minor epithelial layer damage that occurred was observed to be healed in the histological examination of the tissue samples taken on the 14th day (Figure 2). In histological examinations belonging to the propolis group and ABS group, intense angiogenesis and inflammatory cell accumulation were detected, especially in histological sections belonging to the 7th day. It was observed in the tissue samples taken on the 14th day that healing continued, angiogenesis and inflammatory cell accumulation decreased, and wound healing took place rapidly. Histological findings of silk protein and sweetgum oil groups were found to be close to each other. Significantly, it was observed that the healing rates of the tissues taken from the silk protein and sweetgum oil groups on the 7th and 14th days were close and rapid. Among the healing stages, angiogenesis and reepithelization were found to occur quickly, and wound healing was faster in these two groups than in the propolis group and ABS group.

**Figure 3:**
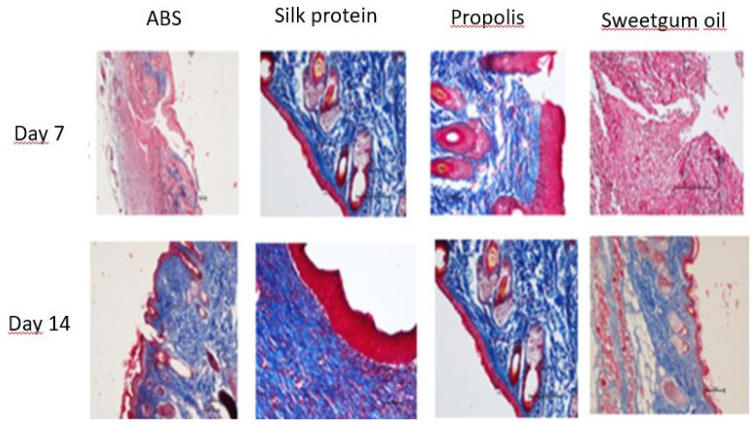
The M.T.T. dye preparations (50 scale bar at 200x magnification)

In histopathological evaluation, reepithelization, granulation, collagen accumulation, inflammatory cell amount, angiogenesis, and ulcer parameters were evaluated (Figure 3). The inflammatory cell density of the propolis group on the 7th day is higher than the other groups. In terms of reepithelization, the sweetgum oil group was at the completed (mature) reepithelialization level in the 7th-day samples. In terms of collagen accumulation on the 7th day, more collagen accumulation was found in the wound samples in the propolis group and the 14th-day samples in the silk protein group compared to the other groups. Intense angiogenesis was observed in the wound samples belonging to the 14th day in the propolis and sweetgum oil groups. In histopathological evaluation, reepithelization, granulation, collagen accumulation, inflammatory cell amount, angiogenesis, and ulcer parameters were evaluated. The inflammatory cell density of the propolis group on the 7th day is higher than the other groups (Table 2).

There was a statistically significant difference between the groups in 7th day EGF, hydroxyproline, IGF, KI-67, 14th day EGF, hydroxyproline, IGF, KI-67 (p < 0.05). Accordingly, the EGF average of the samples taken from the ABS group on the 7th day was significantly higher than the other groups, and the EGF average of the samples taken from the propolis group on the 7th day was significantly higher than that of the sweetgum and Silk protein groups. The mean of EGF measured on the 14th day in the ABS group was significantly higher than in the other groups (p = 0.000). In the measurement of the samples taken on the 14th day from the propolis group, the mean of hydroxyproline was significantly higher than the sweetgum oil group (p = 0.000). The hydroxyproline average of the samples taken on the 14th day from the ABS group was significantly higher than the other groups (p = 0.000). The IGF average on the 7th day in the ABS group was significantly higher than the other groups, and the IGF average on the 7th day in the propolis group was significantly higher than in the sweetgum oil and Silk protein groups (p = 0.000). The 14th day IGF average in the ABS group was significantly higher than the other groups (p = 0.000). The mean of IL-6 on the 14th day in the ABS group was significantly higher than in the propolis and silk protein groups (p = 0.000). The 7th day KI-67 average in the ABS group was significantly higher than the other groups, the 7th day KI-67 average in the propolis group was significantly higher than the sweetgum oil and silk protein groups, and the 7th day KI-67 average in the sweetgum oil group was significantly higher than the silk protein group (p = 0.000). The mean of KI-67 in the 14th-day measurement in the ABS group was substantially higher than in the other groups, the KI-67 average in the 14th-day measure in the sweetgum oil and propolis group was significantly higher than in the silk protein group (p = 0.000) (Table 3).

As a result of the dependent sample t-test applied, there was a statistically significant difference between the times in terms of EGF, hydroxyproline, IGF, IL-6, and KI-67 measurements in the groups (p < 0.05). Accordingly, the mean of EGF, hydroxyproline, IGF, IL-6, and KI-67 measures show a significant decrease.

## 4. Discussion

The wound is a health problem that reduces patients’ quality of life, socially restricts them, increases the loss of workforce and cost of care, and affects the patient and society. Wound care and treatment is a versatile field that concerns many disciplines. For healthy wound healing, treatment and care should be done appropriately. The purpose of the treatment of surgical wounds in the past was to bring the wound lips closer together to heal the wound quickly. Today, however, it has been found that a moist and warm environment created around the wound is more effective in wound healing. This current approach aims to create an ideal environment for wound healing and support healing holistically [2,10]. Dressings containing natural polymers are an issue that keeps up-to-date in terms of the development of new wound treatments due to their biocompatibility, biodegradability, low toxicity, and nonallergenic nature [11].

Many other natural treatment studies for treating cutaneous wounds have been found in the literature in different experimental models. Studies have shown that two main proteins, sericin, and fibroin, which are natural polymers synthesized by epithelial cells in silkworms, are used separately [12,13]. In this study, an extract containing two main types of protein, which is more advantageous in terms of both production technique and cost, was used. Both silk protein cutaneous wound healing supported and shown in operation [11,14]. Safety studies of silk films on rats have confirmed that silk dressings are safe in acute dermal toxicity, acute dermal irritation, and skin sensitization [15,16]. It has been demonstrated that the sericin protein protects against oxidative stress and provides a suitable environment for cell proliferation [17], is effective in the release of inflammatory mediators, but increases the production of collagen without increasing the level of cytokines [18]. In a similar study, in wounds treated with sericin, epidermal thickness and vascularity increased, edema, cellular infiltration, collagen discoloration, and necrosis decreased [19]. A full-thickness skin defect study on rabbits showed that silk fibroin film increased regeneration and effectively reduced average wound healing time compared to commercial wound dressings [20]. In this study, following the literature, it was found that in wounds where silk protein was applied locally, collagen production was higher than sweetgum oil and propolis and wounds healed rapidly.

The sweetgum tree (Liquidambar orientalis), where sweetgum oil is produced, is geographically only seen in the western regions of Turkey and Central America. It is known that the product obtained from the cracks in the trunk of this tree in Turkey has been used in many areas for centuries. It is widely used in public, especially when taken orally, as it positively affects digestive disorders. The effects of this natural substance, which is used folklorically, on wound healing are not fully defined [21–24]. In the study of Altıparmak et al. (2019), Hypericum perforatum, sweetgum oil, and propolis were applied to incisional wounds, and it was reported that these accelerated healing and there was no significant difference between them [25]. Yanık et al. [26] compared the effects of sweetgum oil and silver sulfadiazine in the experimental burn model and found that sweetgum oil was more effective in healing burn wounds than silver sulfadiazine. In this study, it was found that the shrinkage in the wound areas in mice treated with sweetgum oil was faster than in the other groups. In addition, in histopathological examinations, it was observed that angiogenesis was superior to other groups in tissue samples taken on the 14th day.

Propolis is a sticky substance that honey bees produce by mixing various substances they collect from plants with their secretions. Due to a large number of components in propolis content, the combined effect was found to be higher than the sum of the effects of each component alone. The most essential pharmacologically effective compounds in propolis are the flavonoid group (flavones, flavonols, and flavanones) and various phenolics and aromatics [27,28]. In recent studies, it has been reported that propolis, a natural substance, contains vitamins and minerals necessary for the life of living things, increases epithelization, has potent analgesic, immunomodulatory, antioxidant, antitumoral, antibacterial, antifungal, antiviral, antiinflammatory effects, and no side effects [27, 28]. Mouse and rabbit studies have shown that the hydroalcoholic solution of propolis has an antiinflammatory effect after topical injection and oral administration [29]. Propolis contains polyphenols and a wide range of other compounds that can scavenge large amounts of free radicals from our organisms. Propolis has been reported to be effective in dermatology, wound healing, burn and external ulcer treatment, shortening the healing time, increasing wound contraction, and accelerating tissue repair [27,28,31]. In this study, it was observed that reepithelialization of the wounds of mice treated with propolis was superior to other groups. However, it is also a positive feature that the inflammatory cell density in tissue samples taken on the 14th day, which we can describe as the end of the proliferation phase, is less than the other groups.

The effects of ABS, which is produced from 5 different folkloric plants that affect coagulation and is used as a hemorrhage, on wound healing have been investigated in other studies. In their experimental study on rats, Kaya et al. [32] showed that ABS reduced inflammation and wound diameters, increased wound contraction, and tissue fibrosis in rats with burn injury. An experimental study concluded that ABS could be used effectively and safely as a natural product in full-thickness wounds due to its positive histopathological results [33]. Satar et al. [34] obtained better results than the experimental group regarding wound healing time and wound size reduction of ABS application in excisional wounds. In another study, it was observed that ABS application significantly increased fibroblast proliferation [35]. The study of Koluman et al. [36] reported that ABS does not contain heavy metals and pesticides; therefore, ABS application is safe. The same study found that ABS contains various antioxidants (tocotrienols, vitamin E, tryptophan, estriol, galangin, apigenin, oenin, 3,4-divanilylyltetrahydrofuran, thymol, BHA, BHT, lycopene, glycyrrhetinic acid, and tomatin) and these contribute to the pharmacological effects of ABS that have been reported [36]. In this study, IL-6, IGF, EGF, and Ki-67 levels, which are inflammation indicators, were higher than the other groups at the tissue level in wounds treated with ABS. In addition, it was determined that angiogenesis and inflammatory cell density increased more in tissue samples taken at two different times than in other groups. On the other hand, there was no significant difference between the other groups regarding biochemical analysis results and wound closure rate. Based on these data, ABS may contribute to healing in acute wound care, similar to the literature. ABS, which has been shown in many studies to support wound healing and its bleeding-stopping feature [37], can be an alternative in treating acute wounds, especially with open bleeding. However, it is predicted that its use in surgical wound closure will reduce bleeding in the incision and accelerate wound healing [38].

Wound healing, which is an active process, creates characteristic changes in the wound area. These changes, which can be evaluated observationally, guide caregivers in determining the healing phases and not recognizing complications. For example, in the inflammatory phase, while mild edema (exudate) and soft pink skin tissue are seen at the wound site, it is seen that the wound edges approach each other when it comes to the proliferative phase. In the development of infection, symptoms such as purulent discharge, redness, swelling, and pain are observed [1,4]. There were no abnormal signs of wound healing in this study. Hemorrhage, crusting, and any signs of complications were not observed in the wound, and no irritation or allergic reaction developed due to the products used.

Measuring the length and width of the wound area is also a critical evaluation factor in evaluating wound healing. In determining the healed wound condition, the surface area of the wound can be measured using methods such as ultrasound, magnetic resonance, or stereophotometry. Clinically, measurements made with a wound scale and the technique of transferring wound sizes onto acetate are frequently used [5]. This study found that wound surface area values, which are an essential indicator of wound healing, decreased in time in all groups, but wound closure was faster in the sweetgum oil group.

When the literature is reviewed, it is seen that the duration of acute wound healing varies between 8 and 21 days in studies conducted on mice [39,40,41]. Similarly, in this study, the 11–14. it was seen to be completed in the day. Considering the whole closure times of the wounds, it was observed that the average wound healing time was shorter in the silk protein and propolis group compared to the sweetgum oil and ABS group.

Normal and rapid granulation development of tissue is an essential indicator in the wound healing process. Granulation tissue development is evident in open wound healing, and it has been found that it starts 72–96 h after the injury. In experimental studies, it was observed that the fibrin threads of the clot inside the wound directed vertically to the wound surface 3–4 days after the formation of the excision wounds, and approximately six days later, capillaries, fibroblasts, and collagen fibers between the excisional wound took a horizontal structure to the wound surface and brought the wound lips closer to each other [5]. In our study, the inflammatory cell density on the 7th day after wound formation was higher in the propolis group than in the other groups, and low-intensity inflammatory cell infiltration was found in the sweetgum oil group. In the samples on the 14th day, it was observed that the inflammatory cell density in the propolis group was less than in the other groups and more in the sweetgum oil group.

## 5. Conclusions

As a result, in our study, propolis, sweetgum oil, and ABS have shown, with observational and histopathological findings, that silk protein, which has become popular in biotechnology in recent years, contributes to collagen synthesis and accelerates healing. The materials used in the study had different effects on various parameters during the healing stages. For these materials to be used widely in wound treatment, it is necessary to prepare combinations in appropriate doses and make a new research on different wound types. Thanks to the biocompatibility, biodegradability, low toxicity, and nonallergenic nature of these materials, which contain natural polymers, it may be possible to reduce complications in wound care and accelerate healing.

### Limitations of the study

Reduction of the number of animals used during the experiment is an essential factor, in line with the 3R (reduction, replacement, refinement) principles, which should be considered while conducting animal experiments. For this reason, there is no experimental group in the study. Standard wound healing time and wound healing parameters were compared with the results obtained from the experimental groups in this study regarding previous studies.

## Figures and Tables

**Table 1:** Examination of the difference between groups and times in terms of healing time and wound size.

		Sweetgum oil		Propolis		Silk protein		ABS	
		Mean	sd	Mean	sd	Mean	sd	Mean	sd
Healing time		13.11	0.93	12.67	0.5	12.56	0.88	13.44	0.73
Wound size	t_0_	36	0	36	0	36	0	36	0
	t_3_	26.75	7.1	27.22	4.41	27.44	6	32.11	4.01
	t_5_	22.78	3.63	19.56	2.83	19.78	3.49	23.33	4.06
	t_7_	15.11	4.17	13.78	2.91	13.33	3.2	17.33	3.46
	t_10_	3.89^b^	2.42	6.78	2.05	6.56	4.82	9^a^	4.36
	t_14_	0	0	0	0	0	0	0	0
Statistical analysis (F/p)^1^		**412.263/0.000** *		**902.826/0.000** *		**157.592/0.000** *		**83.761/0.000** *	
		**t** **_0_** **>t** **_3_** **>t** **_5_** **>t** **_7_** **>t** **_10_** **>t** **_14_**		**t** **_0_** **>t** **_3_** **>t** **_5_** **>t** **_7_** **>t** **_10_** **>t** **_14_**		**t** **_0_** **>t** **_3_** **>t** **_5_** **>t** **_7_** **>t** **_10_** **>t** **_14_**		**t** **_0_** **>t** **_3_** **>t** **_5_** **>t** **_7_** **>t** **_10_** **>t** **_14_**	

a,b,c,d show the differences between the means of the groups (a = highest mean).

1 Repeated Measures ANOVA test,

* p <0.05

**Table 2:** Investigation of the relationship between groups with reepithelialization, granulation, collagen, inflammatory cell, angiogenesis, and ulcer density.

Histological findings			Sweetgum oil		Propolis		Silk protein		ABS	
			N	%	N	%	N	%	N	%
Reepithelialization	t_1_	Little	0	0	0	0	0	00	6	66.7
		Incomplete but immature or fine	4	44.4	9	100	9	100	3	33.3
		Complete and mature	5	55.6	0	0	0	0	0	0
	t_2_	Incomplete but immature or fine	2	22.2	0	0	2	22.2	6	66.7
		Complete and mature	7	77.8	9	100	7	77.8	3	33.3
Granulation	t_1_	Little	8	88.9	0	0	8	88.9	7	77.8
		Moderate maturation	1	11.1	5	55.6	1	11.1	2	22.2
		Mature	0	0	4	44.4	0	0,0	0	0
	t_2_	Immature	0	0	0	0	4	44.4	5	55.6
		Little	6	66.7	9	100	5	55.6	4	44.4
		Moderate maturation	3	33.3	0	0	0	0	0	0
Collagen	t_1_	Little	7	77.8	0	0	0	0	8	88.9
		Moderately	2	22.2	5	55.6	8	88.9	1	11.1
		Large amount	0	0	4	44.4	1	11.1	0	0
	t_2_	Little	2	22.2	9	100	0	0	0	0
		Moderately	7	77.8	0	0	3	33.3	7	77.8
		Large amount	0	0	0	0	6	66.7	2	22.2
Inflammatory Cell	t_1_	Little	6	66.7	0	0	2	22.2	5	55.6
		Moderately	3	33.3	0	0	7	77.8	4	44.4
		Large amount	0	0	9	100	0	0	0	0
	t_2_	No	0	0	0	0	0	0	4	44.4
		Little	4	44.4	9	100	7	77.8	5	55.6
		Moderately	5	55.6	0	0	2	22.2	0	0
Angiogenesis	t_1_	Less than 5 veins	8	88.9	0	0	8	88.9	0	0
		6–10 veins	1	11.1	3	33.3	1	11.1	3	33.3
		More than 10 veins	0	0	6	66.7	0	0	6	66.7
	t_2_	No	0	0	0	0	5	55.6	4	44.4
		Less than 5 veins	2	22.2	9	100	4	44.4	5	55.6
		6–10 veins	7	77.8	0	0	0	0	0	0
Ulcer	t_1_	Small	0	0	0	0	3	33.3	0	0
		Large ulcer	6	66.7	9	100	5	55.6	7	77.8
		Large or deep ulcer, abscess formation	3	33.3	0	0	1	11.1	2	22.2
	t_2_	No	3	33.3	0	0	3	33.3	2	22.2
		Small	6	66.7	0	0	5	55.6	7	77.8
		Large ulcer	0	0	0	0	1	11.1	0	0
		Large or deep ulcer, abscess formation	0	0	9	100	0	0	0	0

* t1: Examination of tissue samples taken from wounds on the 7th day.

t2: Examination of tissue samples taken from wounds on the 14th day.

**Table 3:** Immunohistochemical analysis IGF, EGF, KI67, IL-6, and hydroxyproline levels investigation of difference between groups and time.

	Sweetgum oil		Propolis		Silk protein		ABS		Statistical analysis (f/p)^1^
	Mean	ss	Mean	ss	Mean	ss	Mean	ss	
EGF (t1)	63^c^	6.16	81.56^b^	6.95	67.56^c^	4.72	97.67^a^	6.12	**60.164/0.000** *
EGF (t2)	37.33^b^	4.85	45.89^b^	5.64	40^b^	3.12	64.78^a^	7.16	**47.433/0.000** *
Statistical analysis (t/p)	**9.879/0.000** * **t1>t2**		**24.868/0.000** * **t1>t2**		**29.268/0.000** * **t1>t2**		**9.098/0.000** * **t1>t2**		
Hydroxyproline (t1)	104.67^b^	9.63	120.67^a^	9.85	114.78	14.73	119.56	10.25	**3.744/0.021** *
Hydroxyproline (t2)	52.22^b^	7.77	66.11^b^	13.54	54.89^b^	12.94	83^a^	5.55	**15.944/0.000** *
Statistical analysis (t/p)	**18.506/0.000** * **t1>t2**		**11.078/0.000** * **t1>t2**		**19.949/0.000** * **t1>t2**		**15.662/0.000** * **t1>t2**		
IGF (t1)	83.22^c^	4.6	101.11^b^	17.24	78.67^c^	3.87	125.56^a^	5.32	**45.074/0.000** *
IGF (t2)	49.78^b^	4.87	59.33^b^	8.72	40.22^b^	4.47	78.78^a^	3.35	**74.602/0.000** *
Statistical analysis (t/p)	**14.02/0.000** * **t1>t2**		**11.146/0.000** * **t1>t2**		**19.834/0.000** * **t1>t2**		**17.432/0.000** * **t1>t2**		
IL-6 (t1)	93.56	7.54	90.33	6.71	90	7.3	97	8.94	1.640/0.200
IL-6 (t2)	55.11	6.19	51.11^b^	5.3	45.78^b^	5.4	63.44^a^	10.79	**9.405/0.000** *
Statistical analysis (t/p)	**18,588/0,000** * **t1>t2**		**22,083/0,000** * **t1>t2**		**47,827/0,000** * **t1>t2**		**14,055/0,000** * **t1>t2**		
KI-67 (t1)	76.33^c^	7.65	105^b^	10.68	63^d^	6.26	123.67^a^	9.07	**92.172/0.000** *
KI-67 (t2)	42.67^b^	7.45	51.67^b^	7.28	26.33^c^	6.93	72.44^a^	7	**64.724/0.000** *
Statistical analysis (t/p)^2^	**22.28/0.000** * **t1>t2**		**29.139/0.000** * **t1>t2**		**16.166/0.000** * **t1>t2**		**13.585/0.000** * **t1>t2**		

a,b,c,d show the differences between the means of the groups (a = highest mean).

1 One-way ANOVA test,

2 Paired sample t-test

* p <0.05

* t1: Examination of tissue samples taken from wounds on the 7th day.

t2: Examination of tissue samples taken from wounds on the 14th day.
